# Supplementary material for: An Enterobacteriaceae bloom in aging animals is restrained by the gut microbiome
Source: bioRxiv. 2023 Jun 14:2023.06.13.544815. Preprint. [Version 1] doi: 10.1101/2023.06.13.544815 (PMC10312681; doi:10.1101/2023.06.13.544815)
Supplement: Supplement 1 [file NIHPP2023.06.13.544815v1-supplement-1.pdf]

bioRxiv preprint doi: https://doi.org/10.1101/2023.06.13.544815; this version posted June 14, 2023. The copyright holder for this preprint (which was not certified by peer review) is the author/funder, who has granted bioRxiv a license to display the preprint in perpetuity. It is made available under aCC-BY 4.0 International license.

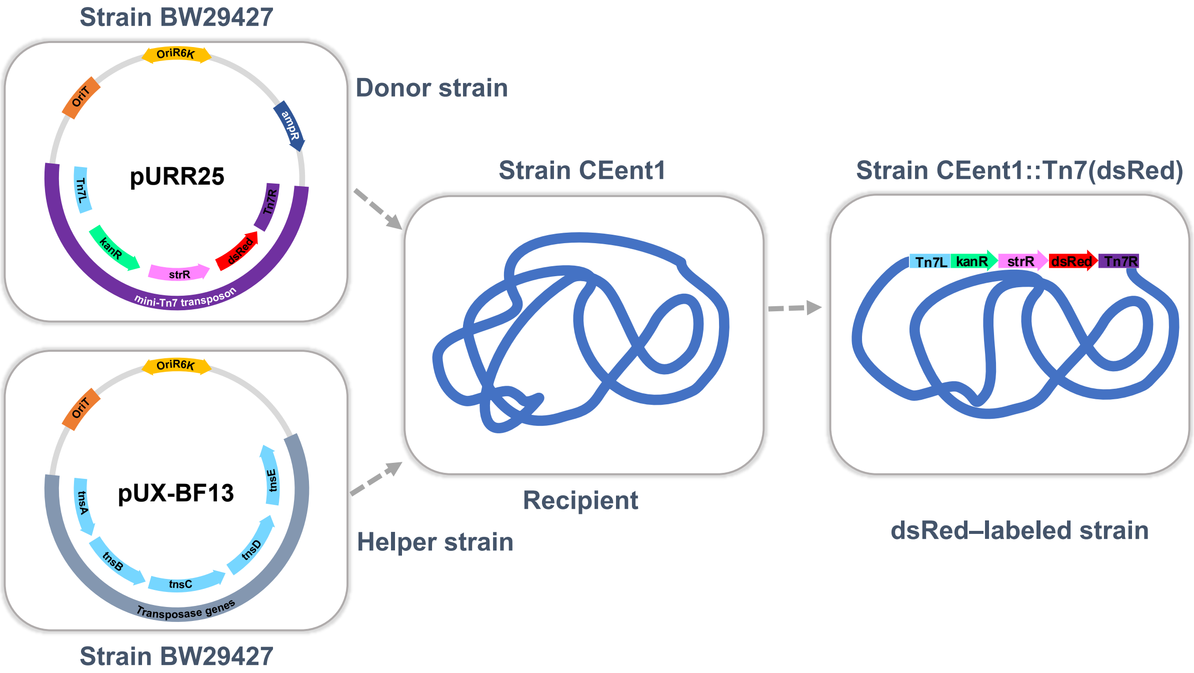

Supplementary Figure 1. Plasmids used in triparental mating to generate dsRed-expressing *Enterobacter hormaechei* CEent1. Shown are maps of the *Tn7-gfp* donor plasmid (pURR25) and the transposase-carrying helper plasmid (pUX-BF13). Tn7R and Tn7L are the sites recognized and cut by the transposase.
